# Alcohol use in opioid agonist treatment

**DOI:** 10.1186/s13722-016-0065-6

**Published:** 2016-12-08

**Authors:** Seonaid Nolan, Jan Klimas, Evan Wood

**Affiliations:** 1 British Columbia Centre for Excellence in HIV/AIDS, St. Paul’s Hospital, 608-1081 Burrard Street, Vancouver, BC V6Z 1Y6 Canada; 2 Department of Medicine, University of British Columbia, St. Paul’s Hospital, 608-1081 Burrard Street, Vancouver, BC V6Z 1Y6 Canada; 3 School of Medicine and Medical Science, University College Dublin, Coombe Healthcare Centre, Dolphins Barn, Dublin, Ireland

**Keywords:** Alcohol use, Alcohol misuse, Alcohol use disorder, Opioid agonist treatment, Methadone, Buprenorphine/naloxone

## Abstract

Alcohol misuse among individuals receiving agonist treatment for an opioid use disorder is common and is associated with significant morbidity and mortality. At present, though substantial research highlights effective strategies for the screening, diagnosis and management of an alcohol or opioid use disorder individually, less is known about how best to care for those with a dual diagnosis especially since common treatments for opioid addiction may be contraindicated in a setting of alcohol use. This review summarizes existing research and characterizes the prevalence, clinical implications and management of alcohol misuse among individuals with opioid addiction. Furthermore, it highlights clinically relevant management strategies in need of future research to advance care for this unique, but important, patient population.

## Background

Approximately one-third of individuals who receive opioid agonist treatment (OAT), such as methadone or buprenorphine/naloxone for the management of an opioid use disorder, also misuse alcohol [1]. Despite alcohol use being a risk factor for fatal overdose among individuals prescribed opioids, as well as being an established risk factor for addiction treatment non-compliance among OAT participants [2–4], little guidance currently exists outlining effective management strategies for this patient population. Consequently, an individual’s alcohol misuse frequently goes undiagnosed and untreated [5–7]. The potential risk for relapse to opioid use, as a result of this missed opportunity, as well as the host of negative consequences that can occur from this or from untreated alcohol misuse is significant among this patient population [8–15]. This review summarizes the existing research of alcohol misuse among OAT participants with a specific focus on prevalence, clinical implications and management. Clinically relevant management strategies in need of future research are additionally highlighted to advance care for this unique, but important, patient population.

## Methods

### Search strategy

This narrative review was based on a literature search using Pubmed and Ovid Medline databases. Keywords used described unhealthy patterns of alcohol use and included: alcohol, alcohol addiction, alcohol misuse, harmful alcohol use, hazardous alcohol use, heavy alcohol use, alcohol abuse, alcohol dependence or alcohol use disorder. These terms were combined with terms referring to OAT including: opioid addiction treatment, OAT, buprenorphine or methadone. Studies written in English were included. Additionally, references for all studies identified through the database search were examined to identify articles that may have been missed. Articles focused on prevalence, clinical implications, screening or management of alcohol misuse among OAT participants were reviewed in detail and are summarized.

## Prevalence

Estimating the prevalence of alcohol misuse among opioid dependent individuals receiving OAT is challenging. Substantial variation exists within the literature among patient populations and treatment settings being studied. Furthermore a lack of standardization pertaining to alcohol misuse terminology and measurement of this is common. In this review, ‘alcohol misuse’ is defined as the consumption of alcohol in a quantity that exceeds low risk for developing an alcohol use disorder as defined by the National Institute on Alcohol Abuse and Alcoholism (i.e. no more than 3 drinks in a single day and no more than 7 drinks per week for women and no more than 4 drinks in a single day and no more than 14 drinks per week for men) and includes both people with ‘risky drinking,’ ‘alcohol abuse or dependence’ and those with an established ‘alcohol use disorder’ [16].

A 2015 review by Soyka et al., estimated one-third of methadone maintenance participants also have problematic alcohol use [1]. Other studies are in agreement with this estimate including a meta-analyses of U.S. clinical trials which demonstrated 38% of individuals seeking treatment for opioid use to have a concurrent alcohol use disorder, as defined by a diagnosis of either alcohol abuse or dependence using criteria from the Diagnostic and Statistical Manual of Mental Disorders (DSM-IV) [9]. Beyond the U.S., data from the British National Treatment Outcome Research Study, a large prospective study of drug users, indicates that at the time of enrolment in a community methadone clinic just over one-third of clients were drinking alcohol above the recommended limits with no statistically significant change observed after 1 year of follow-up [5]. Lastly, a cross-sectional study of current or former heroin users attending primary care for methadone maintenance treatment in Ireland revealed the prevalence of problem alcohol use [as defined by an Alcohol Use Disorder Identification Test (AUDIT) score of >7] to be 35% [17]. Collectively, these data suggest that approximately one-third of opioid addicted individuals in treatment may have concurrent alcohol misuse.

## Interactions

While interactions between alcohol and opioids have previously been described [1, 18], research focused specifically on alcohol in the context of OAT is scarce [19]. Animal studies involving methadone predominate and repeatedly demonstrate an influence of ethanol on methadone metabolism and vice versa [19]. More specifically, among rat subjects, acute ethanol consumption increased peak methadone concentration [20, 21] while chronic ethanol use led to a reduction in peak methadone levels [21–23]. Similarly, acute methadone administration has been shown to decrease the rate of ethanol metabolism (and thus increase blood alcohol levels) [20] whereas chronic methadone use leads to a reduction in blood alcohol levels [24, 25]. Human research focused on this issue is scarce [19]. Clinical observations among individuals who receive methadone maintenance therapy report less of an effect of alcohol [23, 26], more sedation at the time of peak methadone levels as well as more rapid dissipation of methadone’s overall effect resulting in opioid withdrawal symptoms [23]. One study by Lenne et al. [27] did demonstrate a small but significant effect of increased blood alcohol concentration (BAC) among non-opioid study controls compared to those receiving regular OAT. A subsequent study by Clark et al. [19] furthered these findings by demonstrating the interaction between alcohol and opioids to be strongest at the time of peak plasma levels after opioid dosing (i.e. a dose–response relationship) as well as reporting an opioid-specific difference in the magnitude of this interaction (e.g. methadone versus buprenorphine). While these findings support the case for a true pharmacokinetic interaction among humans between alcohol and opioids, the specific site(s) of such interaction requires further study. Furthermore, it should be emphasized that the overall magnitude of the reduction of BAC among individuals receiving OAT in these studies is small (and likely of limited clinical significance) and individuals who receive OAT and consume alcohol will still experience a greater opioid effect due to the combined sedative effect of both substances [19].

## Clinical implications

Knowledge of the potential mechanisms of interaction between OAT and alcohol and their effect on blood levels is of importance, but equally so is determining the clinical significance of these mechanisms.

### Effect of OAT initiation on alcohol consumption

To date, studies investigating the effect of OAT initiation on alcohol consumption among individuals with alcohol misuse and an opioid use disorder are mixed. For example, Caputo et al. [28] demonstrated short term methadone treatment to be associated with a reduction in alcohol levels while long term methadone maintenance therapy resulted in increased alcohol consumption. While an inverse relationship between heroin use and alcohol use has previously been described [29, 30], a recent study found methadone enrolment to have no effect on heavy drinking and may even appear to decrease the initiation of heavy drinking among heroin users [31]. Furthermore, a 12-month longitudinal study of individuals with both heroin addiction and alcohol dependence demonstrated both methadone and buprenorphine to be associated with a reduction in alcohol use, with buprenorphine being more efficacious [32]. Lastly, a recent meta-analyses involving 15 studies showed no clear pattern regarding the effects of OAT on alcohol consumption with 3 studies indicating an increase in alcohol consumption during treatment, 3 studies indicating a decrease in alcohol consumption and 9 studies reporting no change [33].

### Overdose and mortality

Alcohol use has previously been identified to be a risk factor for increased overdose and mortality among individuals receiving OAT [34, 35]. The degree of increased risk conferred overall and according to alcohol consumption patterns however (i.e. hazardous or harmful use compared to an alcohol use disorder) is currently lacking. A cross-sectional study by Zador et al. examined the number and causes of death among participants of a methadone maintenance treatment program in Australia and demonstrated drug-related death to account for the highest proportion of mortality (44%), with alcohol use being cited as the third most common substance of use after benzodiazepines and other opioids [34]. Similarly, a New-York based longitudinal follow-up study of active and discharged methadone patients reported excessive alcohol use (≥4 oz per day for a 3 month period) to be the leading cause of death among active methadone participants (35%) and the second most common cause of death, following complications with opiates, among discharged methadone patients (39%) [35].

Mechanisms driving this process are likely diverse (e.g. suicide attempts, unintentional overdoses involving various substances including benzodiazepines, illicit opioids and OAT, etc.) and not well described, but likely relate to the interactions between alcohol and methadone outlined previously. As such, individuals should routinely be advised of the compounded risk of acute and chronic alcohol consumption while in receipt of OAT and, in particular, of the risk of relapse to illicit opioid use. In addition, during methadone initiation, a period already known to be associated with an increased risk for overdose and mortality [34, 36], concurrent acute ethanol consumption can further compound this risk by increasing CNS and/or respiratory depression [37, 38]. Similarly, though methadone maintenance treatment may lead to lower blood alcohol levels after consumption compared to non-methadone users, one’s overall risk for overdose and mortality is still increased given the combined sedative effect of both methadone and alcohol [26].

### Other clinical outcomes

Beyond increasing one’s risk for overdose and mortality, alcohol misuse among individuals concurrently receiving OAT has been associated with a host of other negative clinical outcomes. Specific to addiction treatment, alcohol misuse has been shown to be risk factor for poor compliance with pharmacotherapy [9] and a predictor for negative treatment outcomes [2–4]. As such, individuals with ongoing alcohol misuse are at an increased risk for a relapse to opioids or other substances [9]. Furthermore, as hepatitis infection is a common comorbidity among opioid dependent individuals with prevalence estimates ranging from 64 to 100% in some cohorts [39–44], chronic alcohol misuse can result in hepatotoxicity and increase an individual’s risk for progression to cirrhosis [11, 13, 15]. Additionally, alcohol misuse can exacerbate psychiatric comorbidities such as anxiety, depression and suicidality, all of which are known to be more common among OAT recipients [8, 10, 12, 45, 46]. Lastly, a study by Sebanjo et al. demonstrated alcohol misuse to be associated with a significant reduction in quality of life and social functioning among methadone maintained individuals [14].

## Screening

Annual screening and brief intervention for alcohol misuse among OAT participants is recommended by clinical guidelines given both its prevalence and potential for a myriad of negative consequences. While the effectiveness of such practices among general populations has shown mixed results [47–49], a significant reduction in alcohol consumption has been observed among methadone maintenance participants in several trials including part of a systematic review [50–54]. More specifically, these studies included participants of both community and designated methadone maintenance clinics in both a European [50, 53] and U.S. [51] setting with the intervention being delivered by either a clinician [50], nurse [51, 53] or trained therapist [51]. Despite this finding, implementation of these interventions among primary care providers of OAT has been slow [55] and remains variable with rates ranging between 2 and 93% [56, 57]. Furthermore, when screening does occur in these settings it is often completed without the use of a validated screening tool [7, 58]. Suggested reasons for these findings identify time restrictions, lack of resources and physician attitudes about the effectiveness of screening and brief intervention for the detection and management of alcohol misuse [52].

Creation of a guideline for alcohol misuse screening and treatment specific for OAT participants has previously been described as a potential solution to mitigate these challenges [59]. Reasons for such a document include: (1) the high prevalence of alcohol misuse among OAT participants, suggesting the need for a more proactive and systematic approach to screening and treatment; (2) consideration for the use of lower thresholds to not only define alcohol misuse but also guide timing for referral to treatment; and (3) the need for involvement of an addiction specialist for severe cases of recurrent or persistent alcohol misuse among this patient population.

While no such dedicated guideline exists in the U.S., a recent clinical guideline was published in Europe and addressed problem alcohol use among substance users who attended primary care (the vast majority for OAT) in Ireland [59]. Screening recommendations from this guideline suggest random, but at least annual, screening for alcohol misuse using AUDIT C (a 3-item version of the Alcohol Use Disorder Identification Test) as an initial screening instrument, with a positive result requiring administration of the full AUDIT. While other diagnostic screening tools (e.g. blood or urine tests, breathalyzer) can be incorporated into the screening process, their utility is limited and should be reserved to either provide pertinent information to a treating physician or help motivate a patient to address their alcohol misuse. A positive screening test for alcohol misuse should be followed up with screening for other substance use and medical comorbidities including hepatitis and other chronic diseases (e.g. cardiac, liver).

## Management

Despite one-third of OAT participants misusing alcohol, treatment for this has widely been ignored [1]. One New York study demonstrated 21% of methadone maintenance patients to misuse alcohol with only 5% being enrolled in outpatient alcohol detox and 7% engaging in psychosocial intervention [6]. A more recent 12-month follow up study among people who use drugs demonstrated little improvement in patterns of drinking among the majority of participants [5]. These findings may be explained by the limited access to alcohol treatment programs that exists for this patient population given many such programs require termination of a patient’s OAT use as a condition of acceptance [6, 60]. To date, though a theoretical risk for over sedation or overdose may exist among OAT participants being treated for alcohol withdrawal, no research to date has clearly quantified the magnitude of this risk or demonstrated any clear interaction between sedative medications used during alcohol detoxification or treatment (e.g. benzodiazepines, barbiturates) and OAT regarding these specific outcomes (though a precipitated opioid withdrawal syndrome has previously been reported with concurrent methadone and phenobarbital administration) [61]. This may relate to the use of these medications within a therapeutic dose range and their administration, which often occurs in a supervised setting. As such, at the present time, no justifiable clinical reason exists to deny entry for treatment of alcohol misuse to an individual that is well established on an OAT program or the need for any modification in dose. Doing so may only increase one’s risk for opioid relapse and the host of negative medical and psychosocial consequences as described above.

Based on the above, all OAT participants identified as having alcohol misuse should be offered treatment. In the acute period, management of alcohol withdrawal in an effective and safe manner is the most important consideration. Unfortunately strategies on how best to accomplish this are lacking in the literature and warrant further study. Validation of risk scoring tools like the Prediction of Alcohol Withdrawal Severity Scale (PAWSS) among this patient population may be of benefit to identify individuals who are at low risk of developing severe, complicated alcohol withdrawal and thus do not require inpatient admission or benzodiazepine therapy for symptom management [62].

Psychosocial interventions for alcohol misuse among OAT participants have previously been described [50, 51, 53]. More specifically, clinician delivered brief intervention was shown to reduce alcohol consumption among OAT participants without alcohol use disorders (AUDIT score <20). Such a treatment approach is recommended for all alcohol misusers identified through screening by the European clinical guidelines previously described [59]. Furthermore a pilot study and randomized controlled trial have identified motivational interviewing to be an effective strategy to reduce alcohol consumption among alcohol misusing methadone maintained participants [51, 53]. Though not specific to OAT participants, psychosocial interventions for alcohol misuse among concurrent substance users have been described in a systematic review [52]. Four studies involving 594 participants evaluated 6 psychosocial interventions through 4 comparison groups: cognitive-behavioral coping skills training versus 12-step facilitation (n = 41) [63], brief intervention versus treatment as usual (n = 110) [64], hepatitis health promotion versus motivational interviewing (n = 256) [51] and brief motivational intervention versus assessment only group (n = 187) [65]. Higher rates of decreased alcohol use were found at 3 and 9 months among the treatment as usual group when compared to brief intervention [64] and more people reduced their alcohol use at 6 months (by 7 or more days in the preceding 30 days) in the brief motivational intervention group compared to control [65]. No other comparisons were found to be statistically significant and because of methodological study differences, no meta-analysis could be performed. Overall the authors were unable to recommend for or against the use of psychosocial interventions for alcohol misuse among concurrent substance users, which is similar to previous findings [66, 67].

While the effectiveness of medications for alcohol relapse prevention including naltrexone and acamprosate has been described among the general population [68, 69], opioid antagonist use among those on OAT is not possible given the effects of naltrexone on OAT and the emergence of precipitated withdrawal. In terms of acamprosate, no studies to date have been conducted among OAT participants who misuse alcohol. The use of disulfiram for reducing heavy alcohol consumption among patients receiving methadone maintenance therapy was evaluated in one randomized double-blind controlled trial [70] with the results showing no significant difference compared to placebo (though the trial was stopped early when sample size targets were not achieved). While two subsequent meta-analyses [71, 72] did demonstrate efficacy with the use of disulfiram when administered in a supervised setting among individuals with alcohol abuse or dependence regarding short term abstinence, days until relapse and number of drinking days, it should be noted that receipt of methadone was an exclusion criteria in one of these studies [71] with the other [72] including only 2 small randomized controlled trials of methadone maintenance patients. Given these findings, there is an urgent need to evaluate the use of such medications, or others used off label for the treatment of alcohol addiction (e.g. gabapentin) [73–75] specifically among OAT participants, as their administration in this setting is a feasible strategy.

Another promising option for this treatment population is extended release naltrexone. Here, while oral naltrexone has not been shown to be superior to placebo in the context of opioid dependence, studies of extended release naltrexone (XR-NTX) have shown promise for the treatment of both alcohol misuse and opioid dependence [76–78]. Prior to initiation with this opioid antagonist, patients are required to have completed opioid detoxification and not be receiving any ongoing opioids (including either methadone or buprenorphine). In settings where XR-NTX is available, this would be an option and its rigorous evaluation in the context of alcohol and opioid poly-substance addiction is warranted.

## Conclusions

Alcohol misuse is common among OAT participants and is associated with a number of adverse outcomes including overdose and mortality. Despite this, the literature suggests that screening and treatment for alcohol misuse among this patient population consistently goes overlooked. To overcome these challenges, future research should focus on the development of strategies to increase rates and frequency of alcohol screening and brief intervention among OAT providers. Guidance for effective alcohol detoxification strategies and an evaluation of acamprosate’s and XR-NTX’s effectiveness for relapse prevention among this patient population is also of importance. Lastly, eliminating barriers for accessing alcohol addiction treatment programs for individuals on OAT is essential as is the integration of alcohol misuse treatment into OAT primary care settings.
